# A multidimensional, efficient, and secure data query based on privacy preservation in vehicular ad hoc networks

**DOI:** 10.1371/journal.pone.0335953

**Published:** 2025-11-26

**Authors:** Xiangmei Zhao, Guofang Dong

**Affiliations:** 1 School of Electrical and Information Technology, Yunnan Minzu University, Kunming, China; 2 Yunnan Key Laboratory of Unmanned Autonomous System, Yunnan Minzu University, Kunming, China; PLOS: Public Library of Science, UNITED KINGDOM OF GREAT BRITAIN AND NORTHERN IRELAND

## Abstract

For vehicular ad hoc networks (VANET) to achieve intelligent transportation applications, efficient and secure data querying is essential. However, sophisticated multidimensional data processing, easy user privacy leaks, and low computational efficiency in resource-constrained contexts are some of the main issues that data querying in VANET environments encounters. To address these issues, this paper proposes an efficient fine-grained data query system (EFDA) based on lightweight masks that allows vehicle users to safely and in real-time query multidimensional traffic data. First, multifaceted data vectors are effectively integrated into a single cipher processing unit using a multidimensional CRT transformation method that counts the number of valid data. Paillier homomorphic encryption and the lightweight region feature masking technique are used to provide safe aggregation while preserving the privacy of the original data. Second, the ECDSA signature is used to ensure source dependability and data integrity. Lastly, to lower system risk and enhance data quality, an effective malicious node monitoring method based on dichotomous recursion and a reputation incentive mechanism based on user feedback is presented. According to security analysis, the EFDA scheme meets the threat model’s specified security requirements for data confidentiality, integrity, source reliability, and identity privacy. According to the performance simulation evaluation, the EFDA system lowers the computation overhead by 85.7% and 90.1% and the communication overhead by 69.1% and 39.2% when compared to the reference scheme. It achieves the balance between privacy protection and query efficiency and validates its viability and efficiency in the resource-constrained in-vehicle network environment.

## Introduction

### Relevant introduction

To meet the demands of intelligent transportation applications [1], data querying in vehicular ad hoc networks (VANET) aims to efficiently, reliably, and promptly retrieve data with strong spatio-temporal correlation from a dynamic distributed network of high-speed moving, resource-constrained vehicles and roadside facilities [2,3]. Abdelatif S et al. [4] proposed a traffic information system architecture based on VANET, and it included fundamental features such as information dissemination, inquiry, and collection. At this point, the majority of research focuses on how to use simple query data in a setting where fast-moving automobiles are present. A VANET-based highway traffic safety information query system is described by Xu et al. [5]. It implements an early and more comprehensive query method by broadcasting query requests and reverse forwarding results between cars. However, in complicated settings where sensitive data, such as vehicle IDs and driving trajectories, are transferred in clear text during the querying process [6] and are traceable, these approaches offer minimal security and privacy protection. Thus, VANETs must develop data query algorithms that meet stringent privacy protection criteria. However, there are still specific difficulties: 1) how to address the issue of data querying security and privacy preservation; 2) how to address the issue of lightweight data querying.

In recent years, researchers have proposed various privacy-preserving enhancements for VANETs. Blockchain technology [7,8] has been introduced to enhance data transparency and tamper-proofness, but its inherent openness and dependence on consensus nodes may bring new risks of privacy leakage. Subsequent studies have enhanced location privacy protection by combining location k-anonymity with steganographic granularity, employing homomorphic encryption [9], and developing secure geographic region authentication schemes that do not require pre-shared keys. Schemes for location-based data aggregation [10,11] have been presented, but multi-domain scenarios are not taken into account. Both using Chinese Remainder Theorem to achieve query privacy protection [12] and adding edge nodes to optimize data indexing have been offered as solutions to the query issues brought on by vast data storage; however, the latter has a considerable communication overhead. Although the combined use of cloud computing and fog computing ensured the sufficiency of computer resources, there is still a significant communication overhead. Early identity-based aggregated signature encryption schemes resolved the issues of secure communication and key escrow. More effective schemes have since been proposed, but they typically rely on expensive bilinear pairing operations, which cause overhead to rise significantly as the number of vehicles increases [13]. The issue of high communication overhead persists despite a recent approach that aims to reduce the amount of bilinear operations [14]. Overall, the ability to balance multidimensional data processing, stringent privacy protection, low overhead, and harmful behavior control is still severely limited by current approaches.

We suggest a novel data querying system intended for use in an in-car network environment, where vehicle users can efficiently query issues like traffic conditions, to overcome the problems above. The primary contributions of this work are as follows:

1. Multidimensional CRT conversion for recognizable quantities: an enhanced CRT technique is put forth for in-vehicle network settings, which captures and identifies the precise number of valid data gathered during the conversion, in addition to effectively converting multidimensional data vectors to large integers for processing.
2. Lightweight mask query scheme (EFDA): this scheme achieves effective fine-grained query matching in the ciphertext domain while rigorously ensuring data confidentiality, integrity, source reliability, and user anonymity. It does this by utilizing mask compression and CRT integration to reduce data redundancy drastically.
3. Effective malicious node control: a binary recursion-based detrimental node tracking technique is put forth, which drastically lowers the localization overhead if verification fails. In the meantime, a reputation incentive system based on user input is intended to recognize, sanction, and remove people who consistently submit dangerous content, while rewarding users who offer accurate information.

The layout of this article is organized as follows. First, “Preparation" introduced the pertinent cryptographic foundations. “System description" described the system model, threat model, and design objectives. Then, “Recognizable number of CRT conversions" proposed a multidimensional CRT transformation method for identifiable quantities. Next, “Efficient fine-grained data query based on lightweight masks (EFDA scheme)" explained the EFDA scheme. “Security analysis" carried out security analysis. “Performance evaluation" focuses on an analysis of computational and communication costs, assessing the efficiency of our proposed scheme. Finally, “Conclusion" presents the conclusion and future work.

### Related work

The security challenge for in-vehicle networks [15,16] has once again become a research hotspot in recent years due to the frequent occurrence of security incidents in these networks. Then, this study discusses the method for the privacy protection problem in in-vehicle networks.

Blockchain is a widely used technology in applications for vehicle networks that ensure anonymity. Li et al. [17] proposed a blockchain-based VANET strategy to address the issues of centralization and mutual mistrust among organizations in the existing VANET. The framework demonstrates superiority in maintaining location and identity privacy. Luo et al. [18] proposed a blockchain-based location privacy protection scheme in VANET, which can protect the location privacy of vehicles during the construction of anonymous and hidden areas. Furthermore, Ilyas et al. [19] suggested a blockchain-based privacy preservation system that improves VANET security while resolving authentication’s unobservability, unlinkability, and efficiency.

VANETs have benefited from the growth of cloud computing. Simultaneously, fog computing has received attention. To guarantee the adequacy of computational resources, numerous researchers have integrated fog computing with cloud computing [20–22]. However, issues with latency and real-time monitoring remain. Gu et al. [23] proposed an effective traceable pavement condition monitoring system based on fog and cloud that preserves privacy and significantly reduces bandwidth and computing resources. Then, to address mutual authentication and anonymity and enable accountable privacy, Rana et al. [24] presented an authentication message exchange strategy using fog-assisted vehicle cloud computing (AME-VCC). Later researchers have proposed schemes like [25–27], but they require bilinear pairing operation, which makes this operation more expensive as the number of cars increases.

Zhou et al. [13] proposed an efficient privacy-preserving data querying scheme (EPDQD). This scheme uses Chinese Remainder Theorem technique to achieve privacy-preserving data querying, but it has a significant communication overhead. The integration of lightweight encryption with edge and fog computing techniques [28,29] addresses the conflict between communication overhead and computational efficiency. Wang et al. [30] introduced a cloud-fog tracking and monitoring scheme (CFTM) that leverages a cloud-fog architecture to facilitate road monitoring, data protection, and malicious node tracking via bilinear pairing. However, the high-cost bilinear operation results in a linear increase in computational overhead proportional to the number of vehicles, posing challenges in meeting real-time requirements. Zhao et al. [31] introduced a privacy-preserving aggregated authentication scheme (PPAAS) that employs aggregated signature technology within a cloud-fog environment to enhance the efficiency of bilinear operations and facilitate conditional privacy protection. The decryption phase continues to depend on the collaborative computation of fog nodes and does not address the issue of fine-grained querying of multidimensional data. Elhabob et al. [32] introduced the Efficient Transmission-Identity-Based Encryption with Chinese Remainder Theorem scheme (ET-IBE-CRF), which employs Chinese Remainder Theorem (CRT) to compress the ciphertext for privacy protection in the cloud. However, it is limited to unidimensional aggregation and lacks a lightweight masking mechanism, resulting in communication overhead that is considerably greater than that of EFDA. Hadabi et al. [33] introduced the Proxy Re-encryption-Identity-Based Signed Privacy Computing Engine scheme (PRE-IBSC-PCE), which integrates proxy re-encryption and identity-based signed secrecy for fine-grained access control. However, it depends on the assumption that fog nodes are entirely trustworthy, leading to complex key management and challenges in tracking malicious users. The EFDA scheme introduces a novel approach to privacy-preserving queries, enhancing overall performance in Telematics through the co-design of multidimensional CRT transformations and lightweight masks. This method minimizes overhead while facilitating efficient cross-dimensional data processing and fine-grained querying. Table 1 provides a detailed comparison of various schemes.

**Table 1:** Option comparison.

Program	multidimensional data	Lightweight	Malicious tracking	Keyless Hosting	Communication Optimization
PPAAS	×	×	√	×	×
CFTM	√	×	√	×	×
ET-IBE-CRF	×	√	×	×	×
PRE-IBE-CRF	√	×	×	×	×
EPDQD	×	√	×	×	×
EFDA	√	√	√	√	√

## Preparation

### Principle of the ECDSA algorithm

The additive cyclic group *E*(*G*_*p*_), the one-way hash function *H*_1_, and the generator *G* with a large prime order *τ* are the public parameters of the Elliptic Curve Digital Signature Algorithm (ECDSA). ECDSA consists of three parts:

1) EKeyGen()→(z,pE): Randomly choose a number as the private key $z\in_{R}Z_{q}^{*}$, then calculate *pE* = *z* ⋅ $G=(x_{1},v_{1})$ as the public key, and finally output the key pair (*z*,*pE*).

2) ESIG(z,m)→(w,d): Take the private key $z\in_{R}Z_{q}^{*}$ and the message *m* as input and choose a random number $r\in_{R}Z_{q}^{*}$, then use the hash function to compute $e=H_{1}(m),(x,v)=r$ ⋅ *G*, and finally $\theta =r^{-1}(e$  +  zw)modτ, where w=xmodτ. Output signature σ=(w,d).

3) EVER(σ,m,pE)→(false||true): Take signature *σ*, message *m* and public key *pE* as input. Then calculate *e* = *H*_1_(*m*), $u_{1}=e/\theta mod\tau$ and $u_{2}=w/\theta mod\tau$. Moreover, calculate $V=u_{1}\cdot G-u_{2}\cdot pE=(x_{y},v_{y})\;and\;y=x_{y}mod\tau$. If y≠w, reject the signature *σ* and output false, otherwise accept the signature *σ* and output true.

### Chinese remainder theorem

Suppose there are *l* pairs of large prime numbers $\left(a_{1},a_{2},...,a_{l}\right)$ and *l* integers $\left(h_{1},h_{2},...,h_{l}\right)$. There exists a unique integer solution *x* satisfying $x\equiv h_{i}moda_{i}$, where 1≤i≤l, can be computed by the following formula *x*:

$$x\equiv h_{1}\alpha_{1}+h_{2}\alpha_{2}+\ldots +h_{l}\alpha_{l}modA$$ (1)

Among them are $A=a_{1}\times a_{2}\times a_{3}\times \ldots \times a_{l}$, $A_{i}=A\cdot a_{i}^{-1}$ and $\alpha_{i}=A_{i}(1/A_{i}moda_{i})$.

CRT also has the following computational properties and can protect data security, assuming that there are two sets of data $\vec{m}=\left\{m^{\left(1\right)},m^{\left(2\right)},\ldots ,m^{\left(l\right)}\right\}$ and $\vec{b}=\left\{b^{\left(1\right)},b^{\left(2\right)},\ldots ,b^{\left(l\right)}\right\}$:

1) Additivity:

$$CRT(\vec{m})+CRT(\vec{b})=(m^{\left(1\right)},m^{\left(2\right)},\ldots ,m^{\left(l\right)})+(b^{\left(1\right)},b^{\left(2\right)},\ldots ,b^{\left(l\right)})\;=(m^{\left(1\right)}+b^{\left(1\right)},m^{\left(2\right)}+b^{\left(2\right)},\ldots ,m^{\left(l\right)}+b^{\left(l\right)})$$ (2)

2) Multiplicability:

$$CRT(\vec{m})+CRT(\vec{b})=(m^{\left(1\right)},m^{\left(2\right)},\ldots ,m^{\left(l\right)})\times (b^{\left(1\right)},b^{\left(2\right)},\ldots ,b^{\left(l\right)})\;=(m^{\left(1\right)}\times b^{\left(1\right)},m^{\left(2\right)}\times b^{\left(2\right)},\ldots ,m^{\left(l\right)}\times b^{\left(l\right)})$$ (3)

### Discrete Logarithm Problem (DLP)

Suppose *G* is a cyclic group of order prime *p* with generator *g* and *f* is an element in *G*.

$$f=g^{x}modp$$ (4)

If the above equation holds, and given a randomized *f*,g∈G, it is difficult for an adversary to compute the value of *x* in probabilistic polynomial time.

## System description

This section describes the various entities within the system, outlining the threat model and design objectives.

### System model

**Fig 1:**
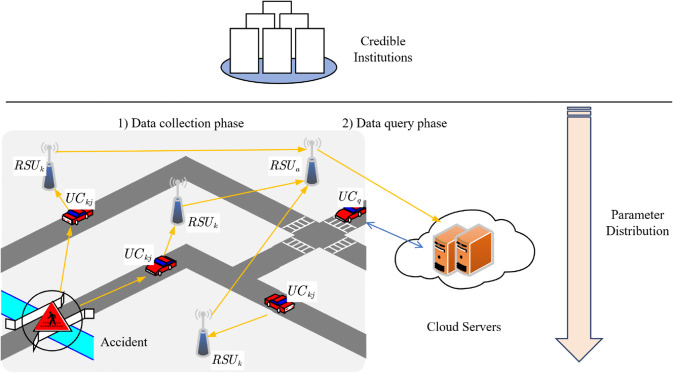
System model.

Fig 1 shows that the system model of this paper is shown. This paper consists of the User’s Car (*UC*_*kj*_), Query Vehicle (*UC*_*q*_), Roadside Unit (*RSU*), Cloud servers (*CS*) and Credible institutions (*CI*) respectively.

*UC*_*kj*_: This entity is the most numerous entity on the road. It is used for the collection of the surrounding situation, providing richer data content for the cloud and facilitating the query of others. It is also important for privacy protection.

*UC*_*q*_: Similar to *UC*_*kj*_, it acts as a consumer who, in order to be informed in advance of issues such as traffic ahead on the road, will initiate a query request to a cloud server as a way of obtaining the data uploaded by *UC*_*kj*_ and scoring the authenticity of the content.

*RSU*: He is a node in the middle between the vehicle and the *CS*, which can help the *CS* to reduce the computational burden. It appears in large numbers in the vehicle network environment. It also has functions such as tracking malicious users in this paper.

*CS*: Cloud servers have enormous arithmetic power and are an important part of the system, not only for storing large amounts of data, but also for monitoring the system for threatening situations. The cloud server decrypts the received ciphertext and will match the relevant data for *UC*_*q*_.

*CI*: This is a similar official, fully trusted entity. It generates the various parameters in the algorithm for the system and sends them to the entities through a secure channel.

### Programmatic flow

**Fig 2:**
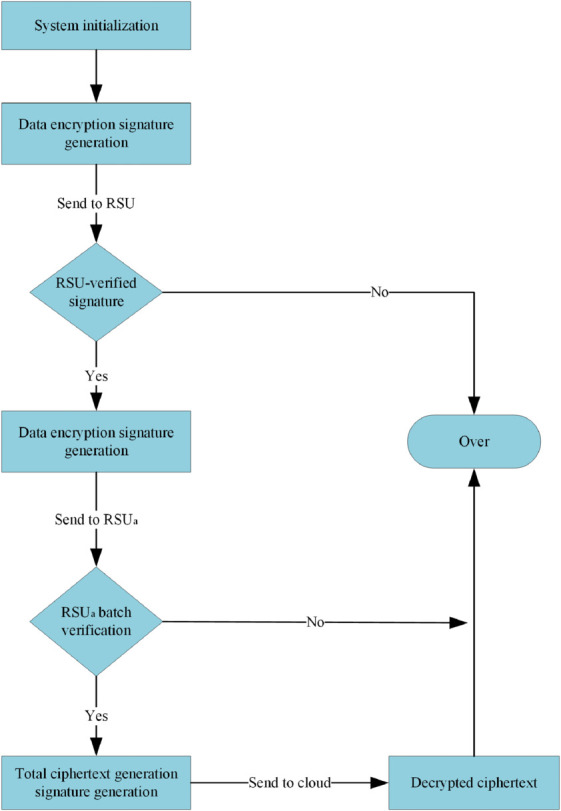
Data upload process.

**Fig 3:**
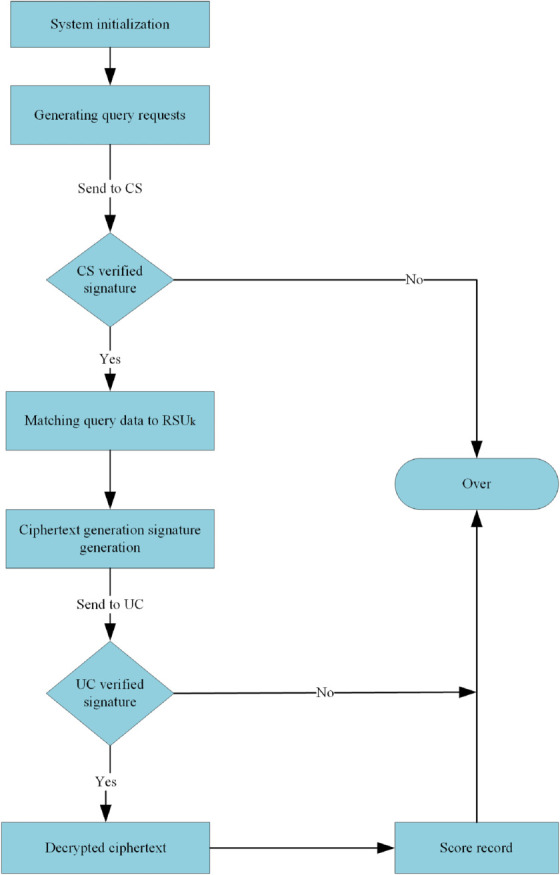
Data query process.

This solution can be subdivided into two parts: data upload and data query, and the flowcharts of the two parts are shown in Fig 2 and Fig 3. The vehicle initiates a query to the cloud server, which first needs to collect data. The vehicle that collects the data first encrypts the data and generates a signature, and then sends it to the *RSU* in its region; the *RSU*, after successfully verifying the signature, aggregates all the ciphertexts it receives into a single message and generates a signature for the message, and then sends it to *RSU*_*a*_ to do the final aggregation operation; *RSU*_*a*_ receives the ciphertexts and verifies the signatures in bulk, and then aggregates all the messages into a total ciphertext and generates a signature for uploading to the cloud server after the signature verification is completed; the cloud server decrypts the ciphertexts after the signature verification is completed. Currently, the cloud server matches the corresponding results concerning the received query and returns them to the querying vehicle.

### Threat modeling

Polynomial time refers to the computational complexity of an algorithm where the time required to complete the task is expressed as a polynomial function of the size of the input. Specifically, if an algorithm runs in polynomial time, its running time can be represented as *O*(*n^k^*), where *n* is the size of the input and *k* is a constant. This attacker 𝒜: Manages a partially semi-honest entity (*RSU*/*CS*) that complies with the protocol while attempting to deduce private information. Possesses access to internal protocol messages but lacks access to the private keys of other entities; engages in active attacks on the communication channel and can access arbitrary messages transmitted within the channel, modify or inject forged messages, and replay historically valid messages.

This section presents six security models derived from the scenario of probabilistic interaction between attacker 𝒜 and challenger *C* within polynomial time: Reliable Source Security Model, Data integrity security model, Privacy preserving security model,Query phase security model,Resisting replay attacks security model and Resistance to Man-in-the-Middle attacks security model. The security boundaries are established through adversarial rules and the challenge game. If attacker 𝒜 exhibits a negligible probability of success in polynomial time across each model, the scenario possesses the corresponding security properties.

Game 1: Reliable Source Security Model

Participants: challenger *C*, attacker 𝒜.

Initialization: *C* runs the system initialization, generates the pseudonym *PID*_*kj*_ for user *UC*_*kj*_, and the corresponding timestamp *t*_*kj*_ and feature mask *Mask*_*kj*_.

Query Phase: 𝒜 can adaptively query to obtain the message tag *MT*_*kj*_ and feature mask *Mask*_*kj*_ of user *UC*_*kj*_.

Challenge Phase: 𝒜 submits the message label $MT_{kj}^{*}$ of the target collection object; *C* generates the legitimate feature identifier ${𝒯}_{kj}^{*}=MT_{kj}^{*}⊕Mask_{kj}$.

Attack phase: 𝒜 outputs the forged feature identifier ${𝒯}_{kj}^{\prime }$, and if ${𝒯}_{kj}^{\prime }={𝒯}_{kj}$, then 𝒜 wins.

The probability of attacker 𝒜 successfully challenging is defined as: $Adv(A)=|Pr[{𝒯}_{kj}^{\prime }={𝒯}_{kj}]-\frac{1}{2}|$

If the probability of attacker 𝒜 winning the challenge within this polynomial time $Adv(A)=|Pr[{𝒯}_{kj}^{\prime }={𝒯}_{kj}]-\frac{1}{2}|$ is negligible, then the scheme satisfies the attribute set commitment verification security property.

Game 2: Data integrity security modeling

Participants: challenger *C*, attacker 𝒜.

Initialization: *C* runs system initialization, generates a homomorphic encryption key ( ) and ECDSA key pair, user pseudonym and *RSU* key ( ), discloses the system parameters and public key ( ), keeps the private key ( ) and the identity of the user *UC*.

Query phase: 𝒜 can adaptively query to obtain the legal ciphertext of the message $C_{kj}=PEnc(pk,M_{kj)}$, the user’s signature of the message $\sigma_{kj}$, the complete packet $Data_{kj}=(\sigma_{kj}||RID_{kj}||PID_{kj}||MT_{kj}||C_{kj}+{𝒯}_{kj}||t_{kj}$.

Challenge phase: 𝒜 submits the target message m^*^ ; *C* generates the legitimate ciphertext $c^{*}=PEnc(pk,m^{*}$, signature $\sigma^{*}$, and outputs $CT^{*}=(c^{*},\sigma^{*},t^{*})$.

Attack phase: 𝒜 outputs the tampered result $CT^{\prime }=(c^{\prime },\sigma^{\prime },t^{\prime })$. If ECDSA. Verify $(pk_{kj},\sigma^{\prime },c^{\prime }||t^{\prime })=true$, then 𝒜 wins.

The probability that attacker 𝒜 succeeds in the challenge is defined as: $Adv_{𝒜}^{INT}(\lambda )=Pr[Awins]$

If the likelihood of attacker 𝒜 succeeding in the challenge in polynomial time $Adv_{𝒜}^{INT}(\lambda )$ is small, then the scheme satisfies the attribute set commitment verification security.

Game 3: Privacy preserving security model

Participants: challenger *C*, attacker 𝒜.

Initialization: *C* generates the system parameters; *C* creates two sets of users,*UC*_0_ and *UC*_1_ with their pseudonyms *P*_0_ and *P*_1_ and feature identifiers *T*_0_ and *T*_1_.

Query phase: 𝒜 can query to obtain the encrypted data of user *UC*, and get the feature identifier $\tau_{i}$ and the dissimilarity value of the mask $MT_{kj}=\tau_{kj}⊕Mask_{kj}$.

Challenge phase: 𝒜 submits two equal-length messages *m*_0_ and *m*_1_; *C* randomly selects b←[0,1], obtains the encrypted data *c*_*b*_ and generates the feature identifier *T*_*b*_.

Attack phase: 𝒜 outputs the guess $b^{\prime }\in [0,1]$, if $b^{\prime }=b$, then 𝒜 wins.

The probability that attacker 𝒜 succeeds in the challenge is defined as: $Adv_{𝒜}^{PRIV}(\lambda )=|Pr[b^{\prime }=b]-\frac{1}{2}|$

If the probability that attacker 𝒜 wins the challenge in this polynomial time $Adv_{𝒜}^{PRIV}(\lambda )$ is negligible, then the scheme satisfies the attribute set commitment verification security.

Game 4: Query phase security model

Participants: Challenger *C*, Attacker 𝒜

Initialization: *C* runs system initialization, generates user *UC*_*q*_’s key *sE*_*q*_, pseudonym *PID*_*q*_, and message tag *CID*.

Query phase: 𝒜 can adaptively query query requests *q*_*u*_ and signatures $\sigma_{q}$, generating query messages $Q=(q_{u}||\sigma_{q}||t_{q})$.

Challenge phase: 𝒜 submits query request $q_{u}^{*}$, *C* generates the result $M_{q}^{*}=\left\{MCI({\vec{m}}_{q}),MT_{kj}\right\}$ to be returned to *UC*_*q*_, encrypts it to obtain $C_{q}^{*}=PEnc(M_{q}^{*},pk_{q})$, and simultaneously generates the signature $\sigma^{*}$, outputting the result $S^{*}\;=\;\left\{\sigma^{*}||M_{q}^{*}||t\right\}$.

Attack phase: 𝒜 outputs the tampered result $S^{`}=\left\{\sigma^{\prime }||M_{q}^{`}||t\right\}$. If ECDSA. Verify $(pk_{q},\sigma^{\prime },M_{q}^{`}||t^{\prime })=true$, then 𝒜 wins.

The probability that attacker 𝒜 succeeds in the challenge is defined as: $Adv_{𝒜}^{INT}(\lambda )=Pr[Awins]$

If the probability that attacker 𝒜 wins the challenge in this polynomial time $Adv_{𝒜}^{INT}(\lambda )$ is negligible, then the scheme satisfies the attribute set commitment verification security.

Game 5: Resisting replay attacks security model

Participants: Challenger 𝒞, Adversary 𝒜

Initialization: 𝒞 runs system initialization, generating keys, pseudonyms, and timestamps for all entities. 𝒞 provides public parameters to 𝒜 and allows 𝒜 to observe previous communication records.

Query Phase: 𝒜 can intercept and record any message $M_{i}=(\sigma_{i},RID,PID,MT,C,t)$ transmitted in previous rounds. 𝒜 can query 𝒞 for the signature of any message under the current or past timestamp.

Challenge phase: 𝒜 selects a previously recorded message *M*  (with timestamp *t* ) and attempts to replay it as a new message to 𝒞.

Attack phase: 𝒜 sends *M*  to 𝒞. 𝒞 verifies the freshness of the signature and timestamp. If 𝒞 accepts *M* , then 𝒜 wins the game.

Adversary Advantage:


$$Adv_{𝒜}(\lambda )=Pr[𝒜\text{ wins}]$$

If $Adv_{𝒜}(\lambda )$ is negligible at security parameter *λ*, then the scheme is resistant to replay attacks.

Game 6: Resistance to Man-in-the-Middle attacks security model

Participants: Challenger 𝒞, adversary 𝒜

Initialization: 𝒞 initializes the system and provides 𝒜 with the public key and parameters. 𝒜 controls the communication channel between 𝒞 and another honest entity (e.g., *RSU* or *UC*).

Query phase: 𝒜 can intercept, modify, or inject messages between 𝒞 and the honest entity. 𝒜 can query 𝒞 for the signature of any message.

Challenge phase: 𝒜 forges a message $M^{\prime }=(\sigma^{\prime },RID^{\prime },PID^{\prime },MT^{\prime },C^{\prime },t^{\prime })$ and attempts to pass it off as a message from the honest entity to 𝒞.

Attack phase: 𝒜 sends $M^{\prime }$ to 𝒞. 𝒞 verifies the signature $\sigma^{\prime }$ using the claimed sender’s public key. If 𝒞 accepts $M^{\prime }$, then 𝒜 wins the game.

Adversary advantage:

$Adv_{𝒜}=Pr[𝒜wins]$

If $Adv_{𝒜}(\lambda )$ is negligible, the scheme is resistant to man-in-the-middle attacks.

### Design objectives

Data confidentiality: Throughtout the process, the data is secured by homomorphic encryption algorithms and no other entity can recover the plaintext from the ciphertext except the *CS*, which can decrypt the data.

Data integrity and reliability: The delivered ciphertext is coupled with a signature algorithm so that the receiver can verify the source of the data and ensure the reliability of the source. Due to the nature of the signature algorithm in this paper, the ciphertext will not be verified successfully if it is incomplete.

Identity privacy: Since the anonymity of the vehicle user in the system is generated for the *CI*, a fully trusted entity, only the *CI* should be able to know the user’s identity and the user acts anonymously in the system.

Resistance to threat attacks: In the system, the scheme guarantees immunity from both threats from internal entities and external adversaries.

## Recognizable number of CRT conversions

### Objectives and core ideas

In in-vehicle network data collection, the data collected by vehicles are usually multidimensional vectors, and it is inefficient to process multiple dimensions directly. This chapter proposes an improved CRT transformation method for efficient integration: the multidimensional data vector ${\vec{m}}_{kj}=\left\{m_{kj}^{\left(1\right)},m_{kj}^{\left(2\right)},\cdots ,m_{kj}^{\left(l\right)}\right\}$ is converted into a single large integer *M*_*kj*_. First of all, use CRT to count and record whether each dimension has collected valid data to get the "quantity" information, then use CRT to compress the multidimensional data vector into a large integer *M*_*kj*_, which is convenient for the subsequent homomorphic encryption and aggregation, and then the cloud gets the large integer after aggregation, and then use CRT to invert the original multidimensional vector and the number of valid data.To comprehend the process, Table 2 lists the relevant annotations.

**Table 2:** Parameter list.

Notation	Definition
*l*	The integer that expresses the dimension of the data.
$\left(a_{1},a_{2},\cdots ,a_{l}\right)$	These are l pairs of large prime numbers, randomly selected by *CI*.
*A*	The product of all dimensional moduli determines the range of values of the large integers. It is the global modulus that must be known for subsequent lossless reduction of each dimension of data.
*A*_*i*_	CRT base coefficient.
$\alpha_{i}$	Modulus inverse coefficient.
*mod*	Modulo operation, i.e., finding the remainder.
*m* and *n*	The number of vehicle users from which the data were collected.
*MCI*()	Encodes a multidimensional integer vector into a large integer via the Chinese Remainder Theorem (CRT).
*ICM*()	Reduce a large integer to a multidimensional vector by the Chinese Remainder Theorem (CRT).

*CI* computes *A*, *A*_*i*_ and $\alpha_{i}$, 1≤i≤l as follows:

$$\left\{ \begin{array}{c} A=a_{1}\times a_{2}\times \cdots \times a_{l}\\ A_{i}=A\cdot a_{i}^{-1}\\ \alpha =A_{i}(\frac{1}{A_{i}}moda_{i}) \end{array}\right.$$ (5)

### Data collection state identification (Algorithm 1)

**Algorithm 1 Results of data collection.**

1: input: Multidimensional data vector

${\vec{m}}_{kj}=\left\{m_{kj}^{\left(1\right)},m_{kj}^{\left(2\right)},\cdots ,m_{kj}^{\left(l\right)}\right\}$

2: output: $f_{kj}^{\left(i\right)}$ and Integer *F*_*kj*_

3: **for** k=1;k≤n;k++; **do**

4:   **for** j=1;j≤m;j++; **do**

5:    **if** $m_{kj}^{\left(i\right)}=0$ **then**

6:     $f_{kj}^{\left(i\right)}\leftarrow 0$

7:    **else**

8:     $f_{kj}^{\left(i\right)}\leftarrow 1$

9:    **end if**

10:   **end for**

11: **end for**

12: $F_{kj}\leftarrow \sum_{i=1}^{l}f_{kj}^{\left(i\right)}$

13: return *F*_*kj*_

Set the vector ${\vec{m}}_{kj}=\left\{m_{kj}^{\left(1\right)},m_{kj}^{\left(2\right)},\cdots ,m_{kj}^{\left(l\right)}\right\}$, where 1≤k≤n and 1≤j≤m are defined. Also, define *F*_*kj*_ to indicate whether the data collection is successful or not, and $f_{kj}^{\left(i\right)}$ to indicate whether the data collection is successful or not for each dimension. As shown in Algorithm 1.

1) If $m_{kj}^{\left(i\right)}=0$, then $f_{kj}^{\left(i\right)}=0$, indicates that the data collection in the *i* dimension fails.

2) If $m_{kj}^{\left(i\right)}\neq 0$, then $f_{kj}^{\left(i\right)}=1$, indicating that the data of dimension *i* is successfully collected.

From this, the exact amount of multidimensional data is $F_{kj}=\sum_{i=1}^{l}f_{kj}^{\left(i\right)}$.

### Multidimensional vector to large integer conversion (Algorithm 2)

Input a multidimensional vector ${\vec{m}}_{kj}=\left\{m_{kj}^{\left(1\right)},m_{kj}^{\left(2\right)},\cdots ,m_{kj}^{\left(l\right)}\right\}$ and modulo inverse coefficients $\alpha_{i}$, operate on each dimension of the multifaceted vector with the corresponding modulo inverse coefficients $\alpha_{i}$, and then aggregate the encrypted multidimensional vector data to get the large integer *M*_*kj*_. For more information, see S1 Appendix.

**Algorithm 2 Multidimensional data vectors are constructed as large integers.**

1: input: Multidimensional data vector

${\vec{m}}_{kj}=\left\{m_{kj}^{\left(1\right)},m_{kj}^{\left(2\right)},\cdots ,m_{kj}^{\left(l\right)}\right\},f_{kj}^{\left(i\right)}$ and $\alpha_{i}$

2: output: Large integer *M*_*kj*_

3: **for** $m_{kj}\leftarrow 0$ **do**

4:   **for** *i* = 1 to *l* **do**

5:    $m_{kj}\leftarrow (m_{kj}+m_{kj}^{\left(i\right)}\cdot \alpha_{i},f_{kj}^{\left(i\right)})$

6:   **end for**

7: **end for**

8: $M_{kj}=\sum_{i=1}^{l}m_{kj}^{\left(i\right)}$

9: return *M*_*kj*_

### Conversion of large integers to multidimensional vectors (Algorithm 3)

Input the obtained large integer *M*_*kj*_ and the large prime previously given by *CI*.The large integer pairs are modulo the prime numbers to get the original input vector ${\vec{m}}_{kj}=\left\{m_{kj}^{\left(1\right)},m_{kj}^{\left(2\right)},\cdots ,m_{kj}^{\left(l\right)}\right\}$. For more information, see S1 Appendix.

**Algorithm 3 : Large integers are constructed as multidimensional data vectors.**

1: input: Large integer *M*_*kj*_ and $\alpha_{i}$

2: output: Multidimensional data vector

${\vec{m}}_{kj}=\left\{m_{kj}^{\left(1\right)},m_{kj}^{\left(2\right)},\cdots ,m_{kj}^{\left(l\right)}\right\}$ and $F_{kj}^{\left(i\right)}$

3: **for** *i* = 1 to *l* **do**

4:   $m_{kj}^{\left(i\right)}=M_{kj}mod\alpha_{i}$

5: **end for**

6: return Multidimensional data vector

${\vec{m}}_{kj}=\left\{m_{kj}^{\left(1\right)},m_{kj}^{\left(2\right)},\cdots ,m_{kj}^{\left(l\right)}\right\}$ and $F_{kj}^{\left(i\right)}$

## Efficient fine-grained data query based on lightweight masks (EFDA scheme)

To comprehend the scheme’s procedure, notations throughout this section are presented in Table 3.

**Table 3:** Parameter list.

Notation	Definition
*RID*_*k*_	Identity label of *RSU*_*k*_
*PID*_*kj*_	The pseudonym of *UC*_*kj*_
*gcd*(,)	Take the greatest common divisor
*Mask*_*kj*_	Lightweight region feature mask for identifying the jth user in the kth RSU region
$H_{1}andH_{2}$	Hash function
*MT*_*kj*_	The “message label” uploaded by the jth user in the kth RSU region
*G*	Generates the meta
*M*	Plaintext
*Q*	User’s query message
*t*_*kj*_	Timestamp
${𝒯}_{kj}$	Feature identifier

**Fig 4:**
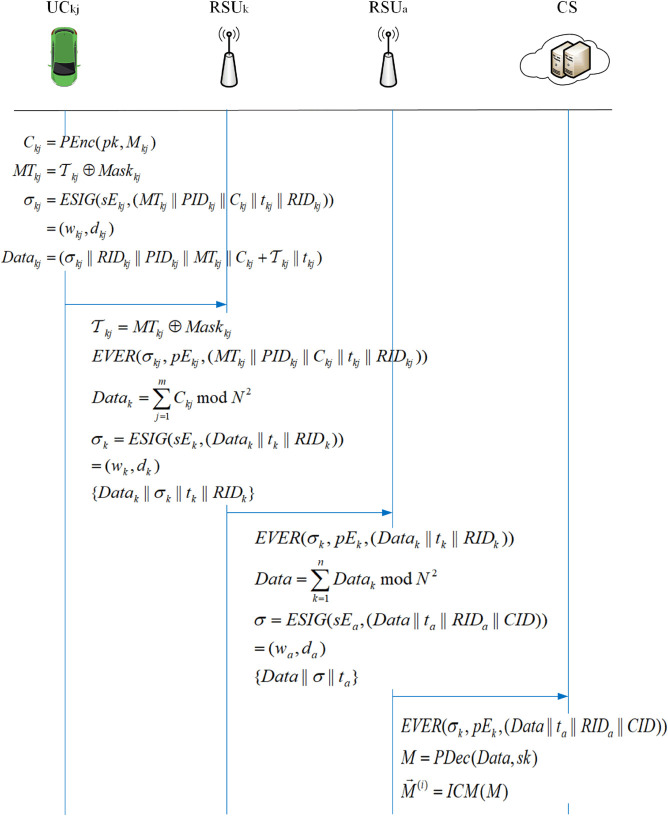
Data acquisition phase.

**Fig 5:**
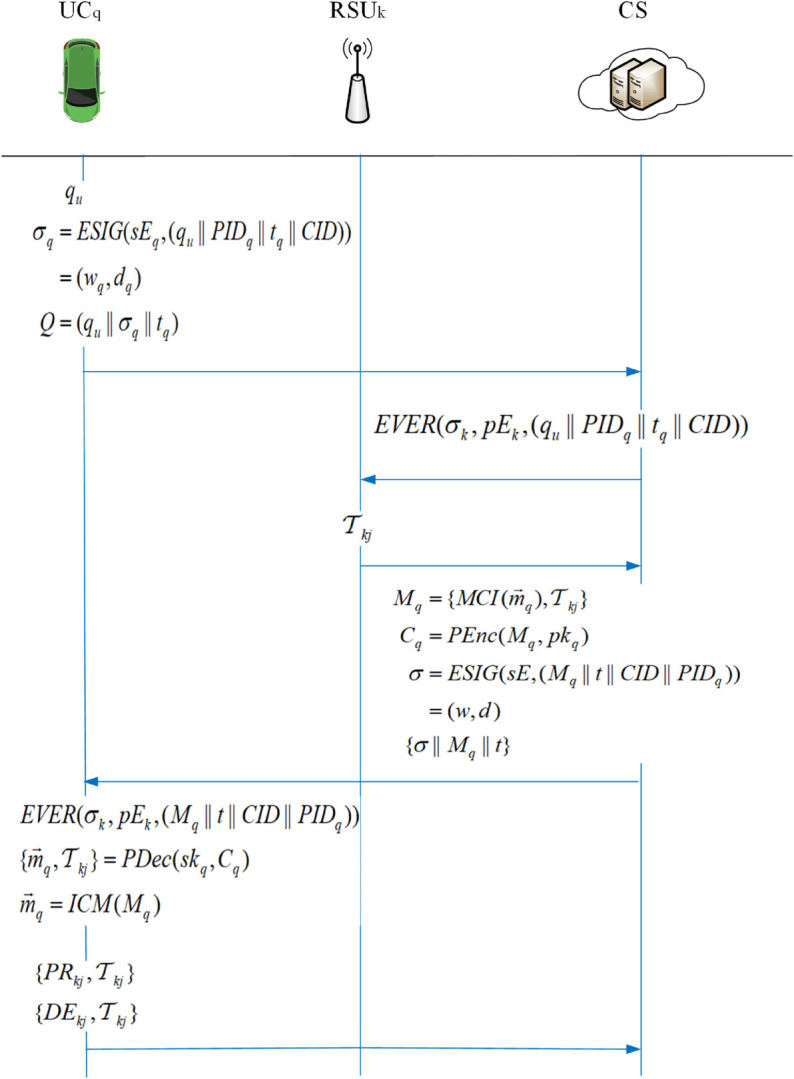
Data query phase.

This section outlines the particulars of an efficient fine-grained data query scheme utilizing lightweight masks. Initially,conduct data collection, encompassing system initialization, the data collection phase utilizing the Paillier encryption algorithm within the framework of homomorphic encryption, the data aggregation phase, and the data reading phase, as illustrated in Fig 4. Upon completion of data collection, users may query based on their requirements, as illustrated in Fig 5. This paper proposes a tracking method and measures for error messages that may arise during the entire phase due to malicious attacks, harmful users, and other hazardous factors.

### System initialization

Given a security parameter *γ* and a dimension *l*. The *CI* generates parameters for homomorphic encryption and ECDSA signatures. The *CI* randomly selects two large prime numbers *p* and *q*, computes *N* = *pq* and λ=lcm(p − 1,q − 1), and defines a function L(μ)=(μ − 1)/N where $\mu =L[(g^{\lambda }modN^{2}){]}^{-1}$. Then it chooses a generator $g\in {\mathbb{Z}}_{N^{2}}^{*}$ and computes $s=r^{N}modN^{2}$, where *r* is a random number and $r\in {\mathbb{Z}}_{N}^{*}$. At the same time, the *CI* generates the public parameters of the ECDSA signature $\left(E(G_{p},q,G,\tau \right)$ and a single hash function $H_{1}:{\left\{0,1\right\}}^{*}⟶Z_{q}^{*}$,$H_{2}:{\left\{0,1\right\}}^{*}⟶{\left\{0,1\right\}}^{p}$.

1) For *CS*, the *CI* generates the public key pk=(N,g) and the private key sk=(μ,λ) for the homomorphic encryption algorithm; in addition, it generates the public key *pE* = *z* ⋅ *G* and the private key *sE* = *z* for the ECDSA signature, where z∈[1,τ−1], and sends (*sk*,*sE*) to the *CS* through a secure channel.

2) For the *RSU*, the *CI* generates the ECDSA public key $pE_{k}=z_{k}\cdot G$ and private key $sE_{k}=z_{k}$, where $z_{k}\in [1,\tau -1]$, and the identity tag *RID*_*k*_ for *RSU*_*k*_,then sends $\left(sE_{k},RID_{k}\right)$ to *RSU*_*k*_ through a secure channel.

3) For *UC*, the *CI* generates the key pair $\left(pk_{kj},sk_{kj}\right)$ for the homomorphic encryption algorithm, the key pair $\left(pE_{kj},sE_{kj}\right)$ for ECDSA, and the pseudonym *PID*_*kj*_ for *UC*_*kj*_, where $pk_{kj}=(N_{kj},g_{kj})$, $sk_{kj}=(\mu_{kj},\lambda_{kj})$, $pE_{kj}=z_{kj}$ ⋅ *G* and $PID_{kj}=H_{2}(ID_{kj}||pk_{kj}||pE_{kj})$. Finally, the *CI* sends $\left(sk_{kj},sE_{kj},PID_{kj}\right)$ to *UC*_*kj*_ and *PID*_*kj*_ through a secure channel to the *RSU* for storage (which is equivalent to the *UC* registering the information with the *RSU*).

In addition, *RSU* randomly generates a feature mask that indicates the location of the area and encrypts it to send to, noting that this mask is lightweight.

### Data collection phase

1) Vehicle *UC*_*kj*_ collects multidimensional data ${\vec{m}}_{kj}=\left\{m_{kj}^{\left(1\right)},m_{kj}^{\left(2\right)},\cdots ,m_{kj}^{\left(l\right)}\right\}$ on its surroundings and itself, and converts these vectors to large integers, i.e., $M_{kj}=MCI(\vec{m_{kj}})$, according to Theorem 1,to facilitate the computation.

2) *UC*_*kj*_ selects a random number $r_{kj}\in Z_{N}^{*}$, where *gcd*(*r*_*kj*_,*N*) = 1. Then, use the public key of CS pk=(N,g) to encrypt the data *M*_*kj*_ as $C_{kj}=PEnc(pk,M_{kj})$, and specifically calculate $C_{kj}=g^{M_{kj}}r_{kj}^{N}modN^{2}$. To reduce the computation overhead and communication overhead, set *g* = *N*  +  1 according to the Paillier cryptographic algorithm, and rewrite the ciphertext *C*_*kj*_ based on the rules of (1 + *N*)^*m*^ = (1  +  *mN*)*modN*^2^:

$$C_{kj}=g^{M_{kj}}r_{kj}^{N}modN^{2}\;=(N+1{)}^{M_{kj}}r_{kj}^{N}modN^{2}\;=(M_{kj}\cdot N+1)\cdot smodN^{2}$$ (6)

3) *UC*_*kj*_ computes the feature identifier ${𝒯}_{kj}=H_{2}(PID_{kj}||t_{kj})$ and obtains $MT_{kj}={𝒯}_{kj}⊕Mask_{kj}$ by mask permutation.

4) *UC*_*kj*_ performs the ECDSA signature algorithm *ESIG*():

$$\sigma_{kj}=ESIG(sE_{kj},(MT_{kj}||PID_{kj}||C_{kj}||t_{kj}||RID_{kj}))\;=(w_{kj},d_{kj})$$ (7)

Where *t*_*kj*_ is the timestamp and $\left(MT_{kj}||PID_{kj}||C_{kj}||t_{kj}||RID_{kj}\right)$ is the message, finally, *UC*_*kj*_ sends the data $Data_{kj}=(\sigma_{kj}||RID_{kj}||PID_{kj}||MT_{kj}||C_{kj}+{𝒯}_{kj}||t_{kj})$ to the RSU.

### Data aggregation phase

After *RSU*_*k*_ receives the data from *UC*_*kj*_, it first checks the validity of its timestamp and verifies the signature by executing the verification algorithm $EVER(\sigma_{kj},pE_{kj},(MT_{kj}||PID_{kj}||C_{kj}||t_{kj}||RID_{kj}))$. After successful verification,*RSU*_*k*_ will aggregate the received data into a new ciphertext *Data*_*k*_. After $\left(RSU_{1},RSU_{2},\cdots ,RSU_{n}\right)$ completes the aggregation of ciphertexts in all regions, it is uniformly sent to the aggregation node *RSU*_*a*_ at the RSU side, where a∉[1,n], and *RSU*_*a*_ will aggregate all ciphertexts into the final ciphertext Data and pass it to the *CS* for the next operation.

1) In order to obtain the message and the corresponding feature identifier of the collection object ${𝒯}_{kj}$, *RSU*_*k*_ decodes *MT*_*kj*_:

$${𝒯}_{kj}=MT_{kj}⊕Mask_{kj}$$ (8)

Then, *RSU*_*k*_ can match the message with a specific identity with each other, which can give accurate results when the user queries and indicate the authenticity of the source.

2) After obtaining ${𝒯}_{kj}$, the received ciphertext can be calculated to get *C*_*kj*_ and then verified. Firstly, *RSU*_*k*_ verifies the signature using the verification algorithm $EVER(\sigma_{kj},pE_{kj},(MT_{kj}||PID_{kj}||C_{kj}||t_{kj}||RID_{kj}))$(in order to save the overhead, batch verification is used).

$$(\sum_{j=1}^{m}y_{kj})mod\tau =(\sum_{j=1}^{m}w_{kj})mod\tau$$ (9)

After successful verification,then accept the ciphertext.

3) Next, *RSU*_*k*_ aggregates the data *Data*_*kj*_ into *Data*_*k*_:

$$Data_{k}=\sum_{j=1}^{m}C_{kj}modN^{2},k=1,2,\cdots ,n$$ (10)

Then *RSU*_*k*_ executes the *ESIG*() algorithm to generate a signature for *Data*_*k*_.

$$\sigma_{k}=ESIG(sE_{k},(Data_{k}||t_{k}||RID_{k}))\;=(w_{k},d_{k})$$ (11)

Finally, *RSU*_*k*_ sends $\left(Data_{k}||\sigma_{k}||t_{k}||RID_{k}\right)$ to *RSU*_*a*_.

4) Based on the assumption that *RSU*_*a*_ is a trusted and secure entity, to minimize the burden on the *CS*, *RSU*_*a*_ aggregates the messages from *RSU*_*k*_. First, check the timestamp of *t*_*k*_ in real time, and then execute the $EVER(\sigma_{k},pE_{k},(Data_{k}||$ $t_{k}||RID_{k}))$ algorithm for verification.

$$(\sum_{k=1}^{n}y_{k})mod\tau =(\sum_{k=1}^{n}w_{k})mod\tau$$ (12)

On success, generate the final aggregated ciphertext Data

$$Data=\sum_{k=1}^{n}Data_{k}modN^{2}$$ (13)

And execute *ESIG*() to generate signature *σ*

$$\sigma =ESIG(sE_{a},(Data||t_{a}||RID_{a}||CID))\;=(w_{a},d_{a})$$ (14)

Finally, send $\left(Data||\sigma ||t_{a}\right)$ to *CS*.

### Data reading phase

When *CS* receives $\left(Data||\sigma ||t_{a}\right)$, it first checks whether *t*_*a*_ is valid or not, and then executes $EVER(\sigma_{k},pE_{k},(Data||t_{a}||RID_{a}||CID))$ algorithm to verify whether $y_{a}mod\tau =w_{a}mod\tau$ is valid or not. If it is valid,it accepts the polymerized ciphertext and proceeds to the next step; otherwise, it rejects it.

CS executes the *PDec*(*Data*,*sk*) algorithm to get the plaintext *M* as follows:

$$M=\frac{L(Data^{\lambda }modN^{2})}{L(g^{\lambda }modN^{2})}\;=\frac{L(Data^{\lambda }modN^{2})}{L((1+N{)}^{\lambda }modN^{2})}\;=L(Data^{\lambda }modN^{2})\cdot \lambda^{-1}$$ (15)

Finally, through Theorem 2, the large integers are reduced to the multidimensional data vector ${\vec{M}}^{\left(i\right)}=ICM(M)$, which can be obtained as $\vec{M}=(M^{\left(1\right)},M^{\left(2\right)},\cdots ,M^{\left(l\right)})$.

### Data query

Vehicles in the in-vehicle network can initiate queries to the *CS* to avoid problems such as upcoming accidents or traffic jams etc. The *CS* quickly matches the results of the questions within the server to the vehicles in response to the queries from the vehicles and returns them to the vehicles.

1) The user $UC_{q},q_{u}\in {𝒵}_{q}^{*}$ initiates a query request to the *CS*, and firstly *UC*_*q*_ computes the signature $\sigma_{q}$:

$$\sigma_{q}=ESIG(sE_{q},(q_{u}||PID_{q}||t_{q}||CID))\;=(w_{q},d_{q})$$ (16)

Then the query message $Q=(q_{u}||\sigma_{q}||t_{q})$ is sent to the *CS*.

2) *CS* receives the query message $Q=(q_{u}||\sigma_{q}||t_{q})$ from *UC*_*q*_ and performs $EVER(\sigma_{k},pE_{k},(q_{u}||PID_{q}||t_{q}||CID))$ to verify of it

$$(\sum_{o=1}^{q}y_{o})mod\tau =(\sum_{o=1}^{q}w_{o})mod\tau$$ (17)

After passing verification, *CS* matches the result ${\vec{m}}_{q}$ with the content of *q*_*u*_. At the same time, *CS* obtains the corresponding flag *MT*_*kj*_ from *RSU*_*k*_, and finally gets the result $M_{q}=(MCI({\vec{m}}_{q}),MT_{kj})$, which is to be returned to *UC*_*q*_, and encrypts it to get $C_{q}=PEnc(M_{q},pk_{q})$, and generates the signature at the same time.

$$\sigma =ESIG(sE,(M_{q}||t||CID||PID_{q}))\;=(w,d)$$ (18)

3) *CS* sends $\left(\sigma ||M_{q}||t\right)$ back to *UC*_*q*_ and decrypts the ciphertext to get the required plaintext $\left({\vec{m}}_{q},MT_{kj})=PDec(sk_{q},C_{q}\right)$, which is ${\vec{m}}_{q}=ICM(M_{q})$, after *UC*_*q*_ checks t and verifies that $y_{a}mod\tau =w_{a}mod\tau$ is valid.

### Tracking and measures against error messages

In vehicle-mounted network systems, the phases of data collection and query are susceptible to threats from malicious nodes or users who may intentionally alter or provide misleading information. Moreover, unstable network environments, such as those characterized by packet loss and interference, can result in signature verification failures and the occurrence of false positives. This section proposes a comprehensive approach to address these challenges, incorporating accurate tracking and reputation incentives.

1) Malicious user tracking: *UC*_*kj*_ sends a message *Data*_*kj*_ to *RSU*_*k*_ at regular intervals, and *RSU*_*k*_ performs BatchVerify on the n messages it receives as a way to ensure the validity of the data and prevent it from being affected by tampered data. When the verification fails, *RSU*_*k*_ launches an efficient binary recursive tracking and localization algorithm to detect the source of the error and find the illegal user. As shown in Algorithm 4.

*RSU*_*k*_ categorizes *j* vehicle users into two groups, *S*1 and *S*2, with each group consisting of *j*/2 users. Threshold determination and secondary validation are essential mechanisms for differentiating between accidental errors and genuine malicious attacks. Upon narrowing the problem to a subset containing a single message, the algorithm does not directly conclude that the user is malicious; instead, it conducts rigorous individual verification of that message.This method can reduce the cost of finding error messages from 2[*j*/2] to [*j*/2]  +  1 times. Then the message is judged by the verification algorithm:

$$\left(\sum_{j=1}^{\left[j/2\right]}y_{kj}\right)mod\tau =\left(\sum_{j=1}^{\left[j/2\right]}w_{kj}\right)mod\tau$$ (19)

If the validation fails, then there is at least one error message in the group; otherwise, the error message is in the group:

$$\left(\sum_{j=[j/2]+1}^{j}\right)y_{kj}mod\tau =\left(\sum_{j=[j/2]+1}^{j}\right)w_{kj}mod\tau$$ (20)

**Algorithm 4 Malicious user tracking with false positive mitigation.**

Input: A set of messages ‘S’ (size ‘n’) from users, Verification algorithm ‘Verify’, Threshold ‘T’ (e.g., T=1 for strict, T=2 for tolerant), User Reputation Scores ‘Rep’ (optional for context).

Output: Identified erroneous message(s) or Suspicious user(s), or indication of potential transient error.

**if** execute BatchVerify(S) == true **then**

return No error detected

**else**

split S into two approximately equal subsets S1 and S2.

**if** execute BatchVerify(S1) == false **then**

**if** ∣S1∣ == 1 **then**

**if** Verify(message in S1) == false **then**

return Identified erroneous message and its sender

*UC*_*suspect*_.

Invoke Reputation Penalty for *UC*_*suspect*_ (See Algorithm 5).

**else**

return Potential transient error or verification anomaly

in subset. Flagged for monitoring

**end if**

**else**

Recursively call Algorithm 4 with Input S1.

**end if**

**else**

**if** ∣S2∣ == 1 **then**

**if** Verify(message in S2) == false **then**

return Identified erroneous message and its sender

*UC*_*suspect*_.

Invoke Reputation Penalty for *UC*_*suspect*_ (See Algorithm 5).

**else**

return Potential transient error or verification anomaly

in subset. Flagged for monitoring

**end if**

**else**

Recursively call Algorithm 4 with Input S2.

**end if**

**end if**

**end if**

Individual validation passes suggest that the initial bulk validation failure could have resulted from a transient network issue or a flaw in the validation logic triggered by a specific combination of benign messages, indicating a false alarm. The system records this event and may opt to attempt a retry later, but does not impose any penalties on the user.

Failure of individual authentication: This indicates that the message is invalid. At this juncture, it is established that either malicious behavior or a node failure has occurred, resulting in the logging of the error and the activation of the reputation penalty mechanism for the user.

The algorithm addresses subset validation failures with multiple messages by recursively splitting and validating, thereby reducing the problem size by half to identify the smallest suspicious group containing the erroneous message. The reduction of reputation value occurs solely upon the confirmation of a malicious message. This mitigates potential harm to a user’s reputation resulting from false positives attributed to network problems. Identifying and tracking instances where individual validations succeed but result in batch failures facilitates subsequent analysis of the system for possible boundary issues or targeted enhancements of the validation algorithm.

2) Measures against malicious messages: the user *UC*_*q*_ acknowledges the queried message $\left(m_{q},MT_{kj}\right)$, and if the message reaches the user’s satisfaction, it affirms the message and returns to the *CS* an affirmation value *PR*_*kj*_ against the source of the message. The user sends $\left(PR_{kj},MT_{kj}\right)$ to the *CS*. Similarly, if the message does not match, a negative value *DE*_*kj*_ is returned, and the user sends $\left(DE_{kj},MT_{kj}\right)$ to the *CS*. As shown in Algorithm 5.

**Algorithm 5 Measures against malicious messages.**

Input: Positive values *PR*_*kj*_, negative values *DE*_*kj*_ and feature identifiers ${𝒯}_{kj}$.

Output: Incentives and removals.

**for** each feedback message (containing ${𝒯}_{kj}$, Score

∈+1(Positive),−1(Negative)) **do**

Identify the corresponding *UC*_*j*_ using ${𝒯}_{kj}$

Update the reputation of *UC*_*j*_: *REP*_*j*_ = *REP*_*j*_ + Score

**end for**

**for** each *UC*_*j*_ **do**

**if** $REP_{j}\geq \theta_{high}$ **then**

Grant incentive

**else if** $REP_{j}\leq \theta_{low}$ **then**

Remove *UC*_*j*_ from system and notify network

**else if** *UC*_*j*_ was flagged by Algorithm 4 (but individual verify

passed) **then**

Increase monitoring level for *UC*_*j*_ for period *T*_*monitor*_

Optionally: Apply a small, temporary reputation penalty

**end if**

**end for**

return (Incentives granted, Users removed, Users under monitoring)

After the *CS* receives an evaluation from a user, it accumulates a score based on *MT*_*kj*_ against the corresponding *UC*_*kj*_. After *PR*_*kj*_ reaching a particular threshold value *δ*, the system grants a specific incentive to the vehicle user; *DE*_*kj*_ also sets a threshold value, if *DE*_*kj*_ reaches the threshold value, the corresponding vehicle user is removed from the system, and the message is announced in the system.

For users *UC*_*kj*_ designated as “suspicious” by Algorithm 4, the system implements enhanced precautions: it intensifies monitoring and applies stricter oversight (e.g., by increasing the sampling rate) of the user’s subsequent messages for a specified duration to determine if the anomaly recurs. A secondary measure involves an optional temporary minor penalty, which allows for a minor and temporary reduction in reputation, considering that the tracking process requires resources and entails risk. This indicates the cost of the incident, albeit to a significantly lesser degree than the penalty associated with confirming malicious behavior. The penalty may be rescinded upon conclusion of the monitoring period, provided there are no issues identified. This approach balances the response to potential risks with fairness towards infrequent errant users.

## Security analysis

This section presents six security models derived from the scenario of probabilistic interaction between attacker 𝒜 and challenger *C* within polynomial time: Reliable Source Security Model, Data integrity security model, Privacy preserving security model,Query phase security model,Resisting replay attacks security model and Resistance to Man-in-the-Middle attacks security model. The security boundaries are established through adversarial rules and the challenge game. If attacker 𝒜 exhibits a negligible probability of success in polynomial time across each model, the scenario possesses the corresponding security properties.

### Source reliability

Game 1: Reliable Source Security Model

Participants: challenger *C*, attacker 𝒜.

Initialization: *C* runs the system initialization, generates the pseudonym *PID*_*kj*_ for user *UC*_*kj*_, and the corresponding timestamp *t*_*kj*_ and feature mask *Mask*_*kj*_.

Query Phase: 𝒜 can adaptively query to obtain the message tag *MT*_*kj*_ and feature mask *Mask*_*kj*_ of user *UC*_*kj*_.

Challenge Phase: 𝒜 submits the message label $MT_{kj}^{*}$ of the target collection object; *C* generates the legitimate feature identifier ${𝒯}_{kj}^{*}=MT_{kj}^{*}⊕Mask_{kj}$.

Attack phase: 𝒜 outputs the forged feature identifier ${𝒯}_{kj}^{\prime }$, and if ${𝒯}_{kj}^{\prime }={𝒯}_{kj}$, then 𝒜 wins.

The probability of attacker 𝒜 successfully challenging is defined as: $Adv(A)=|Pr[{𝒯}_{kj}^{\prime }={𝒯}_{kj}]-\frac{1}{2}|$

Analysis: If attacker 𝒜 successfully forges ${𝒯}_{kj}^{*}$ with non-negligible probability in polynomial time, then the system has a vulnerability in terms of reliable source verification. However, since *Mask*_*kj*_ is randomly generated and confidential, and ${𝒯}_{kj}=H_{2}(PID_{kj}||t_{kj})$ depends on the one-way property of the hash function, the attacker cannot obtain *Mask*_*kj*_ or break the hash function.

### Data integrity

In this system, because of the homomorphic encryption used, in general, the ciphertext reduction does not change the plaintext. A signature accompanies the delivery of the ciphertext, and the source’s signature needs to be verified during the interaction process to ensure the reliability of the source. In this paper, we use ECDSA signatures that have been proven to be secure in [34]. In the data collection phase, signatures are generated by the user, *RSU*_*k*_, and *RSU*_*a*_ during the user to *RSU*_*k*_, *RSU*_*k*_ to *RSU*_*a*_, and *RSU*_*a*_ to *CS* processes, respectively. In the data query phase, the signature is generated by the user and *CS* in the user to *CS* and *CS* to user processes, respectively. An example is given next using the user to the *RSU*_*k*_ process:

*UC*_*kj*_ send $Data_{kj}=(\sigma_{kj}||RID_{kj}||PID_{kj}||MT_{kj}||C_{kj}||t_{kj})$ to *RSU*_*k*_ containing the timestamp *t*_*kj*_ and the signature $\sigma_{kj}$. *RSU*_*k*_ checks the received timestamp and then verifies the signature.

$$\sigma_{kj}=ESIG(sE_{kj},(MT_{kj}||PID_{kj}||C_{kj}||t_{kj}||RID_{kj}))\;=(w_{kj},d_{kj})$$ (21)

The signature contains the ciphertext and other related information; if the ciphertext is incomplete, it will lead to verification failure.

$$(\sum_{j=1}^{m}y_{kj})mod\tau \neq (\sum_{j=1}^{m}w_{kj})mod\tau$$ (22)

If the ciphertext is incomplete, the system will trace the operation. If the verification is successful, it means that the information received is complete and unchanged and the source is also reliable.

### Privacy protection

1) Insider entity threat: In the system, *RSU*_*k*_, *RSU*_*a*_ and *CS* are semi-honest entities who will be interested in the privacy of the source.In this scheme the user operates anonymously throughout. Even though RSU preserved the pseudonym *PID*_*kj*_, the semi-honest entities can not obtain the user’s real identity due to the nature of one-way hashing and the reliability of *CI*. For the feature identifier ${𝒯}_{kj}=H_{2}(PID_{kj}||t_{kj})$, RSUs also cannot get the user identity through it. On the rewards and punishments against the collected data, both the querying user and the *CS* operate on the identity of *PID*_*kj*_ without knowing the true identity of the source. If there is a malicious message or if the system needs to be cleared out, the *CS* will collaborate with the *CI* because the illegal user does not need to be protected.

2) External adversary threat: During the three processes from user to *RSU*_*k*_, *RSU*_*k*_ to *RSU*_*a*_ and *RSU*_*a*_ to *CS*, an external adversary 𝒜 will try to intercept, eavesdrop, or tamper with the information in transit. The homomorphic encryption algorithm used in this paper shows that it’s semantically secure against chosen plaintext attacks in [35].

In the process of user to *RSU*_*k*_, the adversary 𝒜 wants to restore the plaintext $Data_{kj}=(\sigma_{kj}||RID_{kj}||PID_{kj}||MT_{kj}||C_{kj}+{𝒯}_{kj}||t_{kj})$ via. Firstly, 𝒜 needs to find the specific ciphertext *C*_*kj*_, but due to the existence of a mask, the ciphertext becomes *C*_*kj*_  +  ${𝒯}_{kj}$, so 𝒜 needs to obtain ${𝒯}_{kj}$.

$${𝒯}_{kj}=MT_{kj}⊕Mask_{kj}$$ (23)

If 𝒜 wants to get ${𝒯}_{kj}$, 𝒜 needs to know *Mask*_*kj*_, but *Mask*_*kj*_ is hard to get.

In the process of *RSU*_*k*_ to *RSU*_*a*_ and *RSU*_*a*_ to CS, the adversary 𝒜 wants to recover $\sum_{j=1}^{m}C_{kj}$ and $\sum_{k=1}^{n}Data_{k}$ via *Data*_*k*_ and *Data*, then adversary 𝒜 needs to decrypt *PDec*(*Data*_*k*_) and *PDec*(*Data*) in a probabilistic polynomial time. Because of the homomorphic encryption Paillier’s rule, it is necessary to obtain the private key of the algorithm sk=(μ,λ). Because CI is entirely trustworthy, the adversary 𝒜 cannot get the key from CI. So the adversary 𝒜 cannot recover $\sum_{j=1}^{m}C_{kj}$ and $\sum_{k=1}^{n}Data_{k}$ from *Data*_*k*_ and *Data*.

### Security in the query phase

Since the querying vehicle is operating in an anonymized form just like the vehicle collecting the data, neither the internal entity nor the external adversary 𝒜 can know the specific private information. After the querying vehicle receives the encrypted data $\left\{\sigma ||M_{q}||t\right\}$ returned from the *CS*, it verifies the signature according to the rules to prevent risks such as data tampering. Even if 𝒜 gets the ciphertext *M*_*q*_, it cannot recover the data from it because it needs to know the user’s private key in advance, which is difficult within a PPT. That is, given

$$f=g^{x}modp$$ (24)

It is difficult for an adversary to solve for the value of *x* in probabilistic polynomial time. After the vehicle returns the feedback information to the *CS*, the *CS* also calculates according to the feature *MT*_*kj*_. Even if 𝒜 gets *MT*_*kj*_, it can not recover ${𝒯}_{kj}$ because *Mask*_*kj*_ is only known to the vehicle and the *RSU* that collects the data, and it is difficult for 𝒜 to obtain it, which also ensures the privacy of the collecting vehicle.

The query request *Query* = *Enc*(*pk*_*cs*_,*Q*) satisfies IND-CPA, and the response ciphertext *C*_*resp*_ is kept confidential under Paillier. User feedback (*PID*,*Score*), in which *PID* is a pseudonym, satisfies untraceability.

### Prevention of potential attacks

1) Resistance to replay attacks: When a vehicle performs continuous data operations in the same domain, the content of the request verification signature is different each time it sends a request, so it will resist replay attacks. And each time the vehicle performs signature generation, it needs to update the timestamp *t*_*kj*_, which ensures the unpredictability of the update process. The subsequent verification process ensures the freshness of the authentication process because the update of *t*_*kj*_ will cause the previous signature value not to be verified correctly.Therefore, the EFDA protocol is resistant to the replay attacks described above.

2) Resistance to man-in-the-middle attacks: In an open channel, it is possible for an attacker to establish independent associations with the vehicle and the RSU, inducing each other to exchange messages. However, since the attacker cannot get the private key of the vehicle, he cannot forge the correct signature and thus cannot pass the verification. So, the EFDA protocol is able to resist the above mentioned man-in-the-middle attack.

Proof: assumption that the intermediary forged the signature $\sigma^{\prime }$:

$$\sigma^{\prime }=ESIG(sE^{\prime },(MT||PID||C||t||RID{)}^{\prime })\;=(w^{\prime },d^{\prime })$$ (25)

After the forged signature is generated, the middleman sends $\sigma^{\prime }$ to the *RSU*, which performs the verification algorithm *EVER*(). However, the signature is forged at this point, and the verification algorithm is as follows:

$$EVER(\sigma^{\prime },pE,(MT||PID||C||t||RID))$$ (26)

Then calculate *e* = *H*_1_(*m*), $\mu_{1}=e/d^{\prime }mod\tau$ and $\mu_{2}=w/d^{\prime }mod\tau$.

Next, we can compute $V=\mu_{1}\cdot G-\mu_{2}\cdot pE=(x_{y},v_{y})$ and $y=x_{y}mod\tau$.

At this time, according to the signature verification algorithm judgement $y\neq w^{\prime }$, so reject the signature, in other words, the man-in-the-middle attack failed.

## Performance evaluation

This section compares and evaluates the performance of the data collection phase and the data query phase of the proposed scheme. The CFTM scheme [30] and the PPAAS scheme [31] have been selected for comparison. We demonstrate that the proposed EFDA scheme is lightweight and appropriate for the vehicle-mounted network environment. This paper presents experiments conducted on a laptop equipped with an Intel Core i7-5500U CPU at 2.40GHz and 12GB of RAM. The experiments utilize a virtual machine running Ubuntu 18.04, employing bilinearly paired cryptographic libraries (PBCs) and GNU multi-precision algorithms (GMPs), with the programming implemented in C language. Subsequently, we will examine the aspects of communication overhead and computation overhead. Table 4 shows the communication operations cost.

**Table 4:** Communication operations cost.

Operations	Cost(bits)
|C|	100
|ID|	16
|M|	32
|t|	32
|*G*_1_|	320
$\left|{\mathbb{Z}}_{N}^{*}\right|$	512
$\left|{\mathbb{Z}}_{q}^{*}\right|$	160

### Communication overhead

This subsection evaluates the communication overhead of the three schemes: CFTM, PPAAS, and EFDA, focusing on the encryption and decryption stages, respectively. To ensure an equitable comparison, we standardized each communication cost. We applied it equitably, with the units detailed below. |C|=100bits denotes a message,|ID|=16bits denotes an identity or pseudonym,|M|=32bits denotes an identifier,|t|=32bits denotes a timestamp,|*G*_1_| = 320*bits* denotes an element in *G*_1_,$|{\mathbb{Z}}_{N}^{*}|=512bits$ denotes an element in ${\mathbb{Z}}_{N}^{*}$ and $|{\mathbb{Z}}_{q}^{*}|=160bits$ denotes an element in ${\mathbb{Z}}_{q}^{*}$. Table 5 shows the communication overhead of each scheme.

**Table 5:** Communication overhead.

Scheme	Encryption phase	Decryption phase	Total
CFTM	|*ID*| + |*C*| + 6|*G*_1_|	$\left(4n+2)|G_{1}|+n(|{\mathbb{Z}}_{q}^{*}|+|C|+|ID|\right)$	$\left(4n+8)|G_{1}+n|{\mathbb{Z}}_{q}^{*}|+(n+1)(|C|+|ID|\right)$
PPAAS	|*ID*| + |*C*| + 3|*G*_1_|	(2*n* + 1)|*G*_1_| + *n*(|*ID*| + |*C*|)	(2*n* + 4)|*G*_1_| + (*n* + 1)(|*ID*| + |*C*|)
EFDA	$2|ID|+|C|+|t|+|M|+|{\mathbb{Z}}_{N}^{*}|$	$n|ID|+(n+1)(|t|+|{\mathbb{Z}}_{q}^{*}|+|C|)$	$\left(n+2)(|t|+|C|+|ID|)+|M|+|{\mathbb{Z}}_{N}^{*}|+(n+1)|{\mathbb{Z}}_{q}^{*}\right|$

For the sake of comparison, we assume that there is one vehicle user, one RSU, and one cloud server in the system, i.e., *n* = 1. In the CFTM scheme, their communication overhead in the encryption phase is |*ID*| + |*C*| + 6|*G*_1_| = 2036*bits*, their communication overhead in the decryption phase is $6|G_{1}|+|{\mathbb{Z}}_{q}^{*}|+|ID|+|C|=2196bits$, and their total computation overhead is $12|G_{1}|+|{\mathbb{Z}}_{q}^{*}|+2(|ID|+|C|)=4232bits$.

In the PPAAS scheme, their communication overhead in the encryption phase is |*ID*| + |*C*| + 3|*G*_1_| = 1076*bits*, the communication overhead in the decryption phase is 3|*G*_1_| + |*ID*| + |*C*| = 1076*bits*, and the total computation overhead is 6|*G*_1_| + 2(|*ID*| + |*C*|) = 2152*bits*.

**Fig 6:**
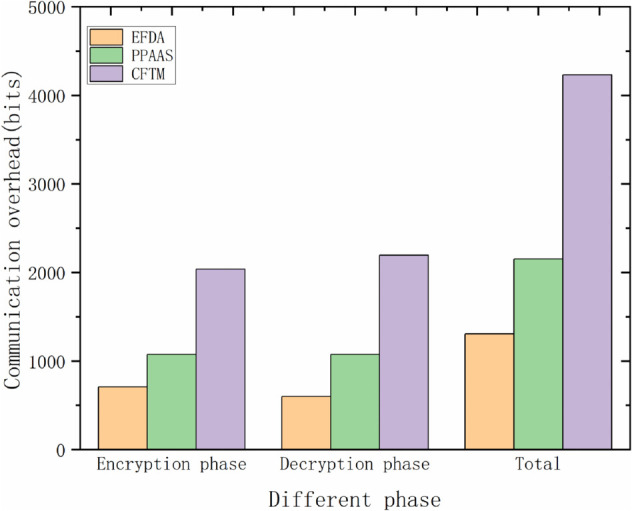
Comparison of communication overhead in different phases of each scheme.

**Fig 7:**
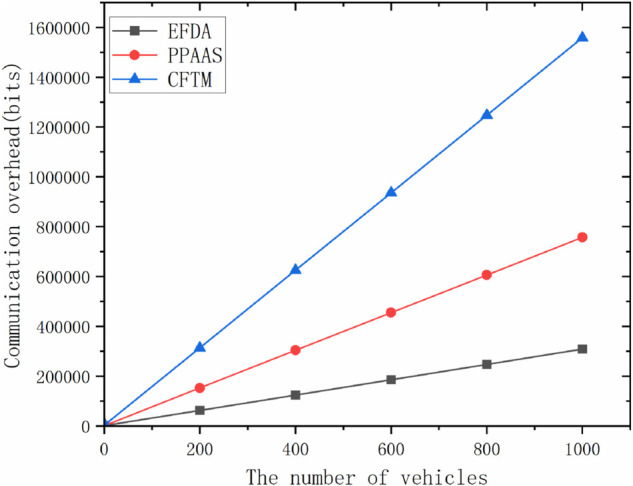
Total communication overhead for each scheme under different numbers of vehicles.

In our EFDA scheme, the vehicle user in the encryption phase sends the message $Data_{kj}=(\sigma_{kj}||RID_{kj}||PID_{kj}||MT_{kj}||C_{kj}+{𝒯}_{kj}||t_{kj})$ to *RSU*_*k*_, which has a communication overhead of $2|ID|+|C|+|t|+|M|+|{\mathbb{Z}}_{N}^{*}|=708bits$; then *RSU*_*k*_ sends the message $\left(Data_{kj}||\sigma_{k}||t_{k}||RID_{k}\right)$ in the region to *RSU*_*a*_, which has a communication overhead of $|ID|+|t|+|{\mathbb{Z}}_{q}^{*}|+|C|=308bits$; and finally the final message $\left(Data||\sigma ||t_{a}\right)$ is sent to the CS by *RSU*_*a*_ which has a communication overhead of $|t|+|{\mathbb{Z}}_{q}^{*}|+|C|=292bits$; and the total computational overhead of this scheme is $3(|ID|+|C|+|t|)+|M|+|{\mathbb{Z}}_{N}^{*}|+2|{\mathbb{Z}}_{q}^{*}|=1308bits$. Fig 6 shows that the comparison of our scheme with CFTM scheme and PPAAS scheme in terms of encryption phase, decryption phase, and total overhead. Fig 7 represents the communication overhead, which changes with the increase of vehicles and RSUs.

It is clear that our scheme has less communication overhead than the CFTM scheme and the PPAAS scheme, both in the encryption and decryption phases, and the burden on the system is within acceptable limits.

### Computational overhead

This subsection contrasts the computational overhead of the three schemes CFTM, PPAAS, and EFDA, examining them in terms of the encryption and decryption phases, respectively. Table 6 illustrates the overheads of the cryptographic operations in question. Serial and different-or operations are also present in the three schemes CFTM, PPAAS, and EFDA. However, their time is negligible, and we therefore uniformly disregard it.

**Table 6:** Operation time.

Operations	Description	Cost(ms)
*T*_*gm*_	Multiplication operation time in *G*	1.429
*T*_*h*_	Hash operation time	0.001
*T*_*p*_	Time using the bilinear pairing operation	12.179
*T*_*m*_	Time with the multiplication operation	0.015
*T*_*e*_	Time with power operations	1.511
*T*_*a*_	Time for addition	0.011

The encryption phase, decryption phase, and overall overhead of the three methods CFTM, PPAAS, and EFDA, are presented in Table 7. We analyze the computational overhead of this scheme in detail.

**Table 7:** Computational overhead.

Scheme	Encryption phase	Decryption phase	Total
CFTM	$6T_{gm}+T_{h}$	$3T_{p}+4nT_{gm}+T_{m}+2nT_{h}$	$3T_{p}+(4n+6)T_{gm}+T_{m}+(2n+1)T_{h}$
PPAAS	6*T*_*gm*_	$5T_{p}+3nT_{gm}+3T_{m}+2nT_{h}$	$5T_{p}+(3n+6)T_{gm}+3T_{m}+2nT_{h}$
EFDA	$T_{gm}+T_{h}+2T_{m}$	$2nT_{a}+(n+2)T_{gm}+T_{e}$	$2nT_{a}+(n+3)T_{gm}+2T_{m}+T_{h}+T_{e}$

**Fig 8:**
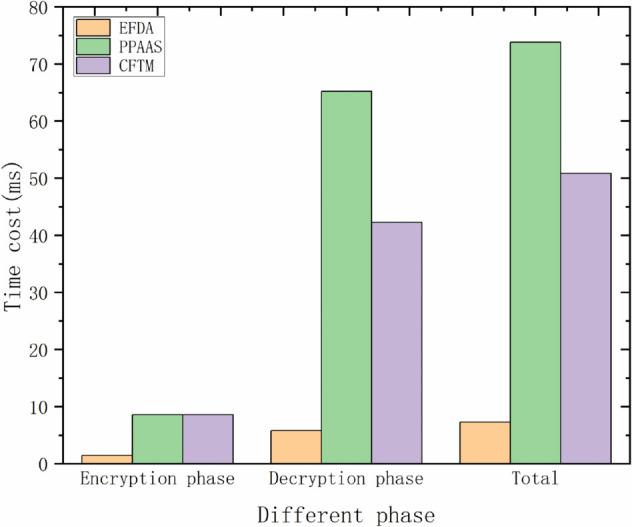
Comparison of computational overhead in different phases of each scheme.

**Fig 9:**
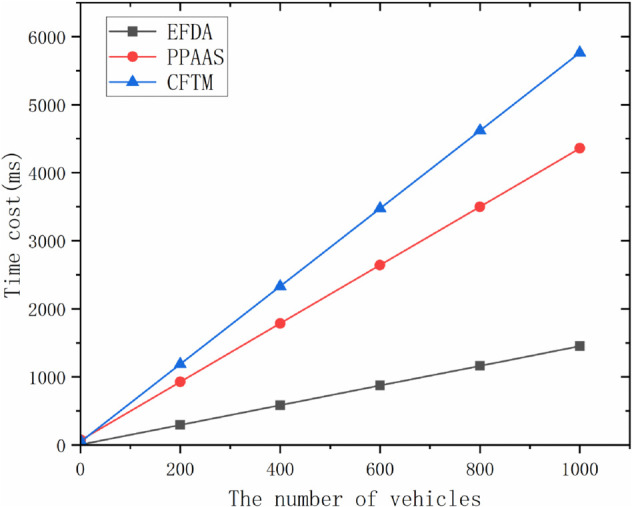
Total computational overhead for each scheme under different numbers of vehicles.

1) Computational overhead of vehicle users: in the data collection phase, each vehicle user will encrypt the collected data $C_{kj}=PEnc(pk,M_{kj})$, because the Paillier encryption we use is modified, and the overhead is optimized, so that it will save much computational overhead. Moreover the vehicle also has to generate a signature and a feature identifier, so the vehicle performs two multiplication operations, one multiplication operation in *G* and one hash computation, i.e., 2*T*_*m*_  +  *T*_*gm*_  +  *T*_*h*_ = 1.64*ms*. It is more efficient than other schemes in the encryption phase.

2) Computational overhead of *RSU*: This layer consists of *RSU*_*k*_ and *RSU*_*a*_. After the vehicle delivers the message to *RSU*_*k*_, it needs to verify its signature. It aggregates the ciphertext into a new message and generates the signature at the same time, and finally sends the latest message to *RSU*_*a*_. Therefore, the computational overhead of *RSU*_*k*_ is *n* ddition operations and *n* multiplication operations in *G*, i.e., $nT_{a}+nT_{gm}$. After receiving a message from *RSU*_*k*_, *RSU*_*a*_ also verifies its signature, the message is aggregated into an aggregated ciphertext, and generate a signature. Note that at the same time, *n* and *RSU*_*a*_ are doing the same operation. Therefore, the computational overhead of *RSU*_*a*_ is *n* addition operations and one multiplication operation in *G*, i.e., $nT_{a}+T_{gm}$.

3) Computational overhead of the cloud server: After the *CS* receives the aggregated ciphertext from *RSU*_*a*_, it verifies the signature and decrypts the ciphertext using the decryption algorithm *PDec*(*Data*,*sk*), which requires one power operation and one multiplication operation in *G*, i.e.,*T*_*e*_  +  *T*_*gm*_ = 2.94*ms*. Thus, our decryption phase and total computational overhead are $2nT_{a}+(n+2)T_{gm}+T_{e}$, $2nT_{a}+(n+3)T_{gm}+T_{e}+2T_{m}+T_{h}$ respectively.

4) CFTM and PPAAS schemes: the computational overheads in the encryption phase of the CFTM scheme and PPAAS scheme are $6T_{gm}+T_{h}$ and 6*T*_*gm*_, respectively; the computational overheads in the decryption phase of the CFTM scheme and PPAAS scheme are $3T_{p}+4nT_{gm}$  +  *T*_*m*_  +  2*nT*_*h*_ and 5*T*_*p*_  +  3*nT*_*gm*_  +  3*T*_*m*_  +  2*nT*_*h*_, respectively; and the total computational overheads of the CFTM scheme and PPAAS scheme are $3T_{p}+(4n+6)T_{gm}+T_{m}+(2n+1)T_{h}$ and $5T_{p}+(3n+6)T_{gm}$  +  $3T_{m}+2nT_{h}$, respectively. Fig 8 shows that the comparison of our scheme with the CFTM scheme and PPAAS scheme in the encryption phase, decryption phase, and total overhead, and Fig 9 represents the computational overhead that changes with the increase of vehicles and RSUs.

In conclusion, our scheme is a lightweight scheme with high efficiency and low computational overhead.

### Query and feedback overhead

**Fig 10:**
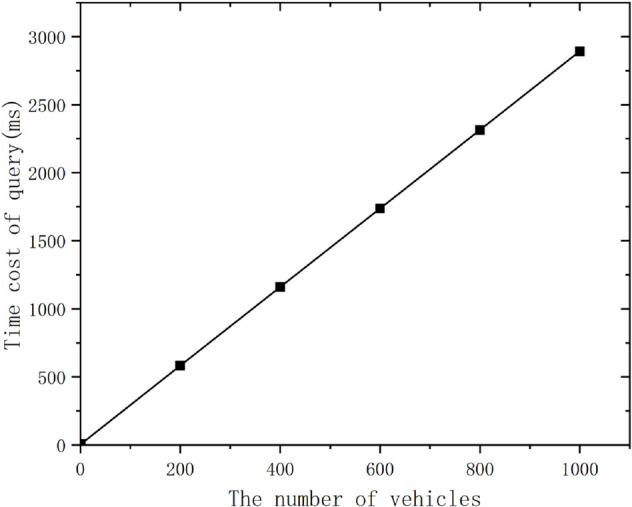
Computational cost of different numbers of vehicles in the query stage.

Computational overhead: this phase requires two multiplication operations in *G* and two multiplication operations in $2T_{gm}+2T_{m}$ in the encryption phase, and one power operation and two multiplication operations in *G* in the decryption phase, i.e., $T_{e}+2T_{gm}$. The total overhead can be seen in Fig 10. Although the overhead increases with the number of vehicles, it is within acceptable limits.

**Fig 11:**
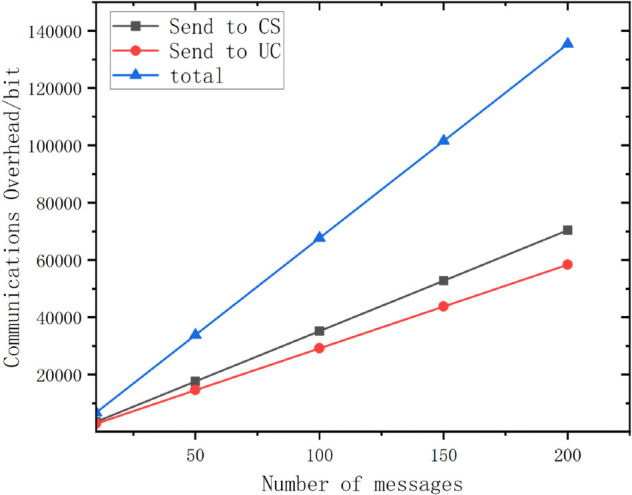
Communication overhead in the query phase for different numbers of messages.

Communication overhead: the querying vehicle sends the message $Q=\left\{q_{u}||\sigma_{q}||t_{q}\right\}$ to the CS, which has the overhead of $2|{\mathbb{Z}}_{q}^{*}|+|t|=352bits$, the CS returns the ciphertext $\left\{M_{q}||\sigma ||t\right\}$ to the querying vehicle which has the overhead of $|C|+|{\mathbb{Z}}_{q}^{*}|+|t|=292bits$, and finally the querying vehicle sends the report $\left(PR_{kj},MT_{kj}\right)$ or $\left(DE_{kj},MT_{kj}\right)$ to the CS which has the overhead of |PR|+|M|=33bits or |DE|+|M|=33bits, of which |PR| and *DE* are 1*bit*, so this scheme is lightweight.The overhead of data query in 1s per unit time is shown in Fig 11.

As can be seen from the figure, the query overhead increases linearly as the number of messages per unit time increases. The overhead is about 13.5KB for processing 200 messages in 1 second, which is within acceptable limits and proves the lightweight nature of this scheme.

### Transmission delay

Transmission delay is a key parameter in evaluating the performance of a network and is analysed in this paper as End-to-End Delay (EED). It is calculated on the basis of the average time taken for data to be received from transmission. For example, in this paper, it is analysed from vehicle user to *RSU* side and *RSU* to *CS* side. The specific expression is given below:

$$TD=\frac{\sum_{k=1}^{n}\sum_{j=1}^{m}(T_{r,kj}-T_{s,kj})}{m_{total}}$$ (27)

Where *T*_*r*,*kj*_ is the number of times the data was received, *T*_*s*,*kj*_ is the number of times the data was sent, and *m*_*total*_ denotes the total number of data. This experiment is conducted on Ubuntu 18.04 to simulate a large vehicle-mounted self-organising network environment with a network coverage area of 3000×2000 square metres. The experimental settings are as follows: The communication distance between the user and the *RSU* is set to find the nearest device within 100 metres, and the simulated data upload time is set to 15 seconds. The experiment consists of two users, A and B, moving at 30km/h and 60km/h respectively.

**Fig 12:**
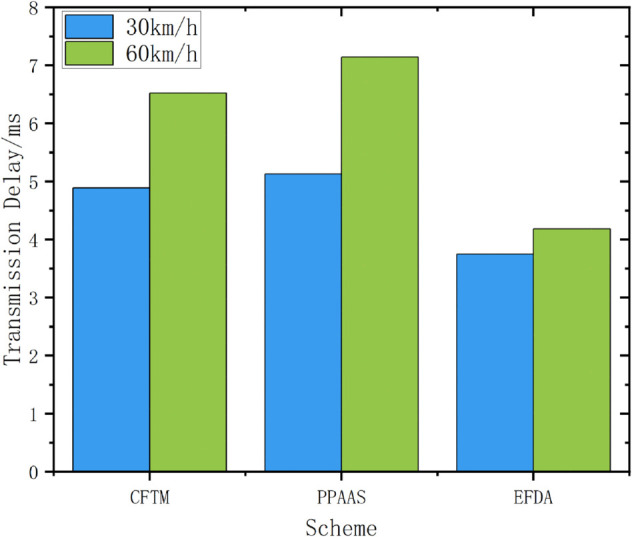
Transmission delay.

As shown in Fig 12, the proposed scheme has lower end-to-end delay than the other schemes, regardless of whether the user speed is 30km/h or 60km/h.

**Fig 13:**
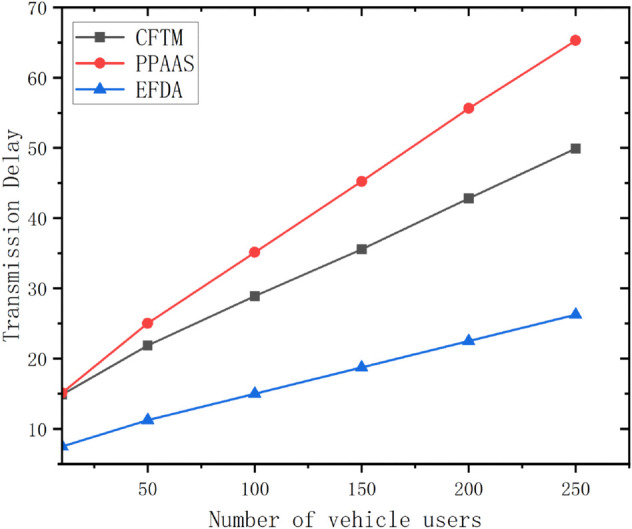
Multi-user transmission delay.

Considering the scalability, we increased the number of vehicle users in the environment with a moving speed of 30km/h and conducted data transmission delay experiments. The results are shown in Fig 13, which proves that the proposed scheme can be deployed in real environments and has a low latency within acceptable limits as the number of vehicle users increases.

## Conclusion

This research offers the efficient fine-grained data query method (EFDA) to address the combined difficulties of security and privacy breaches, as well as the inefficiencies associated with resource-constrained multidimensional data queries in vehicular ad hoc networks (VANET). The primary innovations include: firstly, a multidimensional CRT transformation method developed to ascertain the quantity of valid data, integrated with a lightweight regional feature mask and Paillier homomorphic encryption to facilitate efficient ciphertext aggregation and ensure privacy protection; secondly, the implementation of ECDSA to uphold data integrity and source reliability; lastly, the introduction of an efficient malicious node tracking algorithm employing dichotomous recursion alongside a reputation incentive mechanism to mitigate risk. The security study indicates that EFDA satisfies the criteria for secrecy and integrity while properly balancing privacy protection with query efficiency. Nevertheless, the method is constrained by the robust assumptions of a centralized trust architecture, the latency of Paillier decryption, and the comprehensive coverage of *RSUs*. Future research will concentrate on blockchain-based key management, utilizing blockchain to supplant certain aspects of *CI* to improve system decentralization and mitigate single points of failure.

## Supporting information

S1 File Data. The document contains all the data used in this paper. (XLSX)

S1 Appendix Theorem. The standard definitions of MCI and ICM. (PDF)
