# Supplementary material for: A multidimensional, efficient, and secure data query based on privacy preservation in vehicular ad hoc networks
Source: PLoS One. 2025 Nov 26;20(11):e0335953. doi: 10.1371/journal.pone.0335953 (PMC12654955; doi:10.1371/journal.pone.0335953)
Supplement: S1 Appendix — (PDF) [file pone.0335953.s002.pdf]

Appendix

Theorem 1

Construction of multidimensional data vectors as large integers (MCI): In this paper, we use CRT to construct the multidimensional data vectors as large integers, denoted as $M_{kj} = MCI(\overrightarrow{m}_{kj})$, as shown in Algorithm 2. This aims to make the process more convenient and faster in data processing. The specific expansion formula is as follows:

$$\begin{aligned} M_{kj} &= MCI(\overrightarrow{m}_{kj}) = \sum_{i=1}^{l} m_{kj}^{(i)} \cdot \alpha_i \\ &= m_{kj}^{(1)} \cdot \alpha_1 + m_{kj}^{(2)} \cdot \alpha_2 + \cdots + m_{kj}^{(l)} \cdot \alpha_l \end{aligned}$$

Theorem 2

Construction of large integers into multidimensional data vectors (ICM): In the data reading phase, since the CS recovers aggregated values, it is necessary to use the CRT to convert the large integer aggregated values into multidimensional data vectors, denoted as $m_{kj}^{(i)} = ICM(M_{kj})$, as shown in Algorithm 3. After obtaining $m_{kj}^{(i)} = M_{kj} mod \alpha_i$, the original data $\overrightarrow{m}_{kj} = \left\{ m_{kj}^{(1)}, m_{kj}^{(2)}, \cdots, m_{kj}^{(l)} \right\}$, and the amount of successfully collected data F_{kj} can be recovered.
